# Different Species of Epigeic and Anecic Earthworms Cause Similarly Effective and Beneficial Biocomposting—A Case Study Involving the Pernicious Aquatic Weed Salvinia (*Salvinia molesta*, Mitchell)

**DOI:** 10.3390/life13030720

**Published:** 2023-03-07

**Authors:** Tabassum-Abbasi, Naseer Hussain, Channgam Khamrang, Pratiksha Patnaik, Tasneem Abbasi, Shahid Abbas Abbasi

**Affiliations:** 1 School of Engineering, University of Petroleum and Energy Studies, Dehradun 248007, India; 2 School of Life Sciences, B.S. Abdur Rahman Crescent Institute of Science and Technology, Chennai 600048, India; 3 Centre for Pollution Control & Environmental Engineering, Pondicherry University, Chinnakalapet, Puducherry 605014, India

**Keywords:** salvinia, *Eisenia andrei*, *Perionyx sansibaricus*, *Lumbricus rubillus*, *Drawida willsi*, vermicomposting, biofertilizer

## Abstract

As reported recently by the present authors, vermicomposting by the epigeic earthworm *Eisenia fetida* transforms the highly ligninous and allelopathic aquatic weed salvinia (*Salvinia molesta*) into a benign organic fertilizer. The present study was carried out with four other earthworm species, including three epigeic species of different sizes and phytophagic habits: *Eisenia andrei, Lumbricus rubillus,* and *Perionyx sansibaricus*. One anecic species, with geophytophagous habits, was also explored for comparison: *Drawida willsi*. The objective was to see whether the type of salvinia transformation caused by *E. fetida* is a general phenomenon or whether there are significant differences in the nature of biocomposts generated by different earthworm species. Accordingly, the characteristics of the biocomposts separately generated by each of the six species mentioned above were assessed with UV-visible spectrophotometry, Fourier-transform infrared spectrometry, differential scanning calorimetry, thermogravimetry, and scanning electron microscopy. The studies reveal that, with minor variations, the biocomposting by all four species was able to remove the intransigence of salvinia and impart plant/soil-friendly attributes to it in substantial measures. All the findings obtained with different techniques corroborated each other in arriving at this conclusion. Hence, it can be said that, in general, biocomposting by earthworms takes away the toxicity of pernicious weeds such as salvinia, converting them into plant-friendly and soil-friendly biofertilizers.

## 1. Introduction

### 1.1. Earthworms as Biocomposters

Among the animals whose biocomposting of leaf litter, other plant debris, and animal droppings makes a decisive impact on Earth’s biogeochemical cycles is the earthworm. The extent of the beneficial impact that earthworm-based biocomposting has on the physical and chemical properties of soil rivals the similarly stellar role played by two other types of animals: ants and termites. Together, the three fragment, move, physicochemically modify, and rejuvenate most of the Earth’s soil, earning the nickname ‘soil-engineers’ [1].

However, ants and termites are highly eu-social animals, possessing very rigid and hierarchical social structure; the majority of their population comprises a ‘working class’, which forms the bottom of a pyramid-like social structure in which the privilege of reproducing is concentrated on a single individual at the top: the queen [2]. This means that, barring very few exceptions, the workers of ants and termites cannot breed, and thus no reactor based on the use of worker termites or ants can function beyond the small lifetime of the workers introduced in it. Additionally, no culture of any species of ants or termites can be developed in isolation from the concerned colony [3].

In contrast, earthworms can breed in captivity, and cultures ranging from very small in size to very large can be easily developed and maintained. Within earthworm-based reactors, or vermireactors, earthworms breed easily—with new generations coming up as the previous ones reach their end. For this reason, vermireactors can potentially be operated indefinitely and at scales varying from very small to very large.

### 1.2. Challenges Associated with the Controlled Biocomposting of Phytomass by Earthworms

Despite the advantages mentioned above, commercial-level vermicomposting has so far been largely confined to using animal manure for feedstock. Whereas earthworms predominantly biocompost plant debris in nature, they have not been utilized to a significant extent in controlled vermicomposting of phytomass-based waste. The prime reason for this [4] has been the inability of the earthworms to survive in conventional vermireactors if fed with phytomass instead of animal manure. Due to the large depth-to-surface-area ratio of conventional vermireactors and the resultant development of anaerobic pockets and/or layers in those vermireactors, earthworms get stressed and die. Maintaining uniform moisture levels in such vermireactors is also very difficult because most of the moisture drains down to the lower parts of the reactor. This also stresses the earthworms, who have to contend with either dry zones or excessively wet ones. Similar problems with conventional vermireactors also arise when using manure feed, but they do so to a much lesser degree than when they use phytomass feed. Attempts to get around these problems have led past researchers to add 50% or more of animal manure to the phytomass feed [5]. Some authors have blended the phytomass with 50% or more of animal manure, then composted it, and then attempted the vermicomposting of the compost. However, all such attempts had common drawbacks. Firstly, animal manure is in much shorter supply than phytomass, since weeds contribute billions of tons of phytomass every year. Secondly, animal manure has competing uses, unlike most weeds. For this reason, animal manure fetches a higher price than weeds. Hence, if phytomass-based vermireactors have to depend on animal manure for 50% of their capacity, it will seriously limit their ability to process the available phytomass. Thirdly, adding the slow and fairly expensive processing step of prior composting, attempted by some authors, can make the overall vermicompost production process commercially unviable.

### 1.3. The High-Rate Vermicomposting Paradigm

To solve the problems associated with vermicomposting phytomass, S.A. Abbasi and his co-workers have advanced the high-rate vermicomposting paradigm [4]. With it, and the technology developed to implement it [6,7,8], they have succeeded in vermicomposting a large variety of phytomass, including even toxic weeds such as parthenium [9] and recalcitrant xerophytes such as prosopis [10]. More significantly, they have not only achieved direct and sustained vermicomposting of phytomass, without the need for any pre-processing or additives, but they have also succeeded in doing so at rates 3–4 times faster than what had been achievable with pre-existing, conventional vermicomposting systems. They have also shown that after the earthworms in high-rate vermireactors slowly adapt to single-species phytomass feed, they begin to grow and reproduce well—with the second and the third generations living in the vermireactors showing increasing vermicomposting efficiency [11]. This continues until a peak performance is achieved and then maintained in subsequent generations.

### 1.4. The Present Work

As reported recently by these authors [12], the free-floating aquatic weed salvinia (*Salvinia molesta*)—which is one of the 100 most invasive of all species [13] and arguably one of the biggest colonizers of inland water courses—is highly ligninous. For this reason, salvinia is even more difficult to biodegrade than most terrestrial weeds. It is also allelopathic, owing to the presence of biomolecules in it that repel other species of plants and suppress their growth [9]. However, these authors have shown [12] that when the earthworm *Eisenia fetida* vermicomposts salvinia, the latter gets dramatically transformed into a plant-friendly and soil-friendly biofertilizer. This finding has made it possible to utilize the very large quantities of salvinia biomass, estimated at billions of tons per annum, that are generated annually in the wetlands of the world [14,15].

At present, due to the absence of any viable options for utilizing it, this salvinia biomass degrades aerobically or anaerobically in water bodies, depending on whether the degradation occurs in the water’s oxygenated or anoxic zones. While aerobic degradation outputs CO_2_, anaerobic degradation generates a 1:3 mixture of CO_2_ and CH_4_, in addition to N_2_O. Since all of these are global warming gases (CH_4_ and N_2_O being 86 and 264 times more potent than CO_2_ over a 10-year time horizon, respectively [16]), salvinia biodegradation contributes massively to the problem of global warming. Infestation of wetlands by salvinia also harms the wetlands in several other ways [11,14,15], with major adverse effects on water storage capacity, water quality, biodiversity, fisheries, and the recreational value of the wetlands. Salvinia promotes the growth of disease vectors, breaks water currents, and inhibits navigation. The combined effect of all these happenings is catastrophic. For these reasons, a process such as the one discussed in the present study, which has the potential to utilize all available salvinia for organic fertilizer generation, may have a major beneficial impact in most of the tropical and sub-tropical regions of the world. Other innovative biotechnological options for utilizing salvinia have included anaerobic digestion [17,18], nanoparticle synthesis [19], and use as a source of catalyst for microbial fuel cells [20] and biomolecules [11]. However, no such option has shown potential for utilizing large quantities of salvinia, nor have any of the options indicated likely commercial viability.

Before large-scale vermicomposting of salvinia is attempted, it is necessary to check whether the nature and extent of bioremediation achieved by *E. fetida* can be replicated by other epigeic or anecic earthworm species. The present study was designed to seek the answer to this question and assess the role of four earthworm species—three epigeic (phytophagous) earthworms and one anecic (geophytophagous) earthworm─in vermicomposting salvinia. The novelty of this work stems from the following aspects:

(a) It is based on a process which *directly* and *rapidly* vermicomposts a weed, in contrast to most of the previously reported processes [4,5,21,22,23] which necessitated some form of pre-treatment, besides manure supplementation to the extent of 40% or more of the vermireactor feed. As explained in detail by Abbasi et al. [4,6], this necessity for pre-treatment and manure supplementation makes the processes costly and complicated from engineering and economical points of view. It is for this reason that vermicomposting of phytomass is not conducted at large scale (in comparison to the commercially viable manure vermicomposting), despite the fact that much larger quantities of phytomass are available for vermicomposting than of animal manure.
(b) The weed studied—salvinia—is exceptionally ligninous and hardy. The success of the process with such a weed would indicate that the process will be even more effective with less problematic substrates.
(c) It explores the comparative effect of as many as four different earthworm species (including three epigeic species and an anecic species), of different average masses and sizes, on the vermicompost generated from the same substrate (salvinia), under identical conditions. Prior to this work, only one or two epigeic species had been studied in this manner, with no clarity on how vermicomposts generated by epigeics compare with vermicomposts generated by anecics.

## 2. Experimental Section

### 2.1. General

Vermicomposts of salvinia were generated separately, utilizing each of the four earthworm species: *Eisenia andrei*, *Perionyx sansibaricus, Lumbricus rubellus,* and *Drawida willsi.* For this, rectangular fiberglass vermireactors were utilized. They were 40 cm in area and had a height of 12 cm. At the bottom of each reactor, a moist, 3 mm thick jute cloth was laid out to hold moisture. Next, 4 kg of salvinia was fed to the reactors in the form of whole plants which had been air-dried. No chopping, grinding, or pre-treatment of any kind was performed on the salvinia.

In each of the reactors, 25 individual earthworms were released per kg of salvinia feed. These were randomly selected for the purpose from the cow dung-fed cultures maintained in the authors’ laboratories. Care was exercised to include only those of the randomly picked earthworms that had attained adulthood and were robust and healthy. The contents of all vermireactors were kept 60 ± 10% moist by sprinkling required quantities of water on them daily.

After the biocomposting began, the vermicast was generated in a granular, seed-like form, distinguishable from the rest of the reactor contents [4]. It was harvested for quantification as vermicompost by separating it from undigested substrate, using a harvesting machine previously designed and reported by S.A. Abbasi and co-workers [7,24]. Harvesting was performed once per month, and the vermireactors were kept running by charging them with quantities of fresh salvinia equivalent to the quantities consumed. In this manner, a solids retention time (SRT) of 30 days was observed, and continuous reactor operation was maintained, despite the process being essentially a pulse-fed batch process. The vermicast production was 1–1.5 kg during the first two months because the earthworms, who had been cultured on cow dung, took time to adapt to the new feed. In subsequent months it gradually rose to 1.8–2.2 kg per reactor. From the vermicast produced by such reactors, roughly 5 g each of three samples were randomly selected and pooled. Further samples for analytical work were drawn from the well-homogenized versions of these pools.

### 2.2. Analytical Studies

Samples of the substrate and vermicast/vermicompost were assessed for their carbon and nitrogen content using a Varion EL Cube model autoanalyzer. To generate the FTIR spectra of salvinia and its biocompost, the samples were oven dried at 110 °C and finely ground before being homogenized with potassium bromide (KBr) powder, using a mortar and pestle. They were then shaped into pellets at a pressure of about 1 MPa. A Nicolet iS50 FTIR instrument was used to record the spectra of the pellets over a range of wavelengths encompassing 4000–400 cm^−o^, scanned at a speed of 0.5 cm s^−1^.

For the thermal analysis, comprising thermogravimetry and differential scanning calorimetry (DSC), a composite, model SDT Q600 V20.9 Build 20 thermal analyzer was deployed. The scanning was performed under a non-oxidizing nitrogen atmosphere. It covered a temperature range of 30–1000 °C, with heating rates of 10 °C/minute and 5 °C/minute for thermogravimetry and DSC, respectively. The system was kept under a manometric pressure of 101 KPa throughout.

To obtain the surface morphology of salvinia and its vermicast, scanning electron micrographs were recorded with gold-spluttered samples, using a Hitachi S-3400N instrument.

## 3. Results and Discussion

### 3.1. C:N Ratio

**Figure 1:**
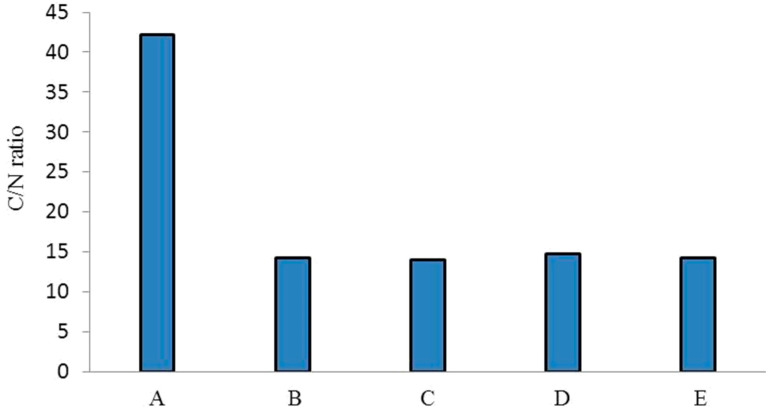
C:N ratios of salvinia (A) and of the vermicomposts derived from *E. Andrei* (B), *P. sansibaricus* (C), *L. rubillus* (D), and *D. willsi* (E).

The C:N ratios of biocomposts produced by different earthworm species compared to those of the parent substrate (salvinia) are shown in Figure 1. There is almost a threefold difference between salvinia and its vermicomposts in terms of C:N ratio—whereas salvinia has a ratio of 42:1, its vermicomposts have a ratio of less than 15:1. Kumar et al. [25] have reported similar results, recording a 23% reduction in the C:N value of vermicomposts derived from flower waste using the earthworm species *Eisenia fetida.* Furthermore, Amouei et al. [26] observed 48%, 36%, and 43% decreases in the C:N ratio of household solid waste, raw biological sludge, and chemical sludge, respectively, in vermicomposts produced by the earthworm species *Eisenia fetida* over a period of 70 days. The extent of this transformation in the relative concentrations of carbon and nitrogen is exceedingly important because it has direct ramifications for the vermicasts’ fertilizer value.

Whereas a biocompost possessing a C:N ratio of <20 is designated as adequately stabilized for promoting agriculture, a C:N ratio of <15 (14.5) is regarded to be ‘highly desirable’ in a fertilizer.

Vermicomposting is thus seen to achieve a very high degree of stabilization, turning salvinia—which has no fertilizer value—into a highly potent nitrogen-rich manure. The trend in the extent of stabilization achieved by the four species of earthworm is *P. sansibaricus* (14) > *D*. *willsi* (14.2) > *E. andrei* (14.3) > *L. rubillus* (14.8). However, the range of difference is very narrow, indicating that all four species are equally effective.

The main reason why vermicomposting brings such a drastic change in the relative concentrations of carbon and nitrogen is evidently the decomposition of the organic carbon present in salvinia by earthworm-induced biodegradation [27,28]. It releases inorganic nitrogen, possibly in addition to other nutrients such as phosphorous and trace elements hitherto locked in the organic matrices. The rise in nitrogen concentration, causing a reduction in the C:N ratio, may also be aided and abetted by the nitrogen-rich mucus that the earthworms release into the vermicompost, as previously suggested by Bhat et al. [29].

### 3.2. FTIR Spectrometry

**Figure 2:**
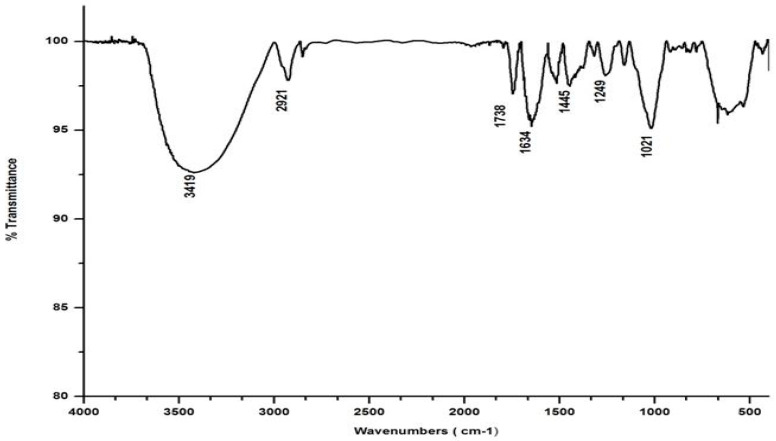
FTIR spectrum of salvinia.

Salvinia’s FTIR spectrum, reproduced in Figure 2, shows a broad band in the wavelength range 3000–3500 cm^−1^, indicating that several organic acids, phenols, and alcohols are present in salvinia [30]. Following this, another band appears at 2921 cm^−1,^ due to stretching in aliphatic C-H groups [31]. This kind of stretching is reportedly manifested by fatty acids and/or lipids [29]. This band is succeeded by a maximum at 1738 cm ^−1^, which may be associated with the -COOH group and the stretching vibrations of esters that may be present in substances of ligneous origin [32]. The next pronounced maximum, at 1634 cm^−1^, appears to have arisen due to aromatic C=C vibrations [25]. Another maximum occurs lower down, around 1520 cm^−1^, which may be due to skeletal vibrations occurring in holocellulose [33]. Evidently, salvinia contains exceptionally high phenol levels, which causes its allelopathy, while the high lignin content makes it hardy and difficult to degrade.

The FTIR also shows peaks at 1445 and 1249 cm^−1^. These peaks are likely associated with the -OCH3 and C-O stretching of lignin and phenol molecules, respectively [32]. Further down the spectrum, a peak at 1021 cm^−1^ was detected, possibly attributable to the C-O stretch of molecules containing polysaccharides and holocellulose [34].

**Figure 3:**
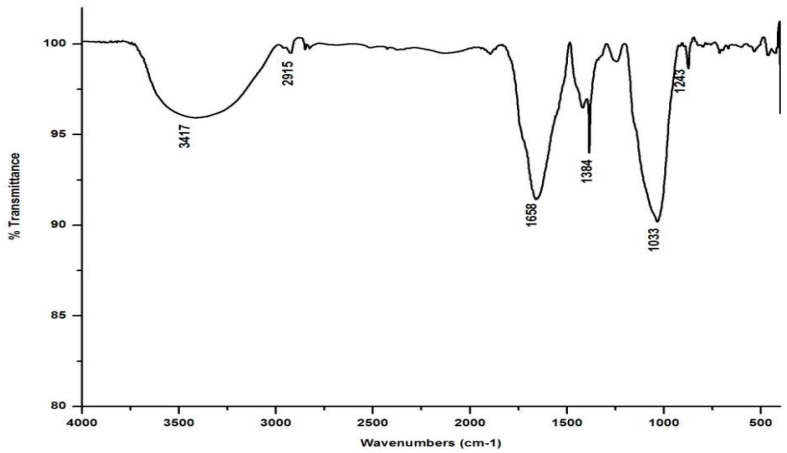
FTIR spectrum of the vermicompost generated from salvinia by *E. andrei*.

**Figure 4:**
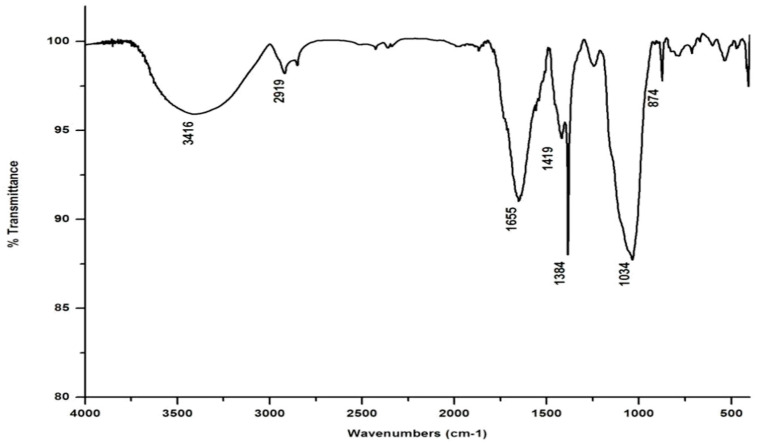
FTIR spectrum of the vermicompost generated from salvinia by *P. sansibaricus*.

**Figure 5:**
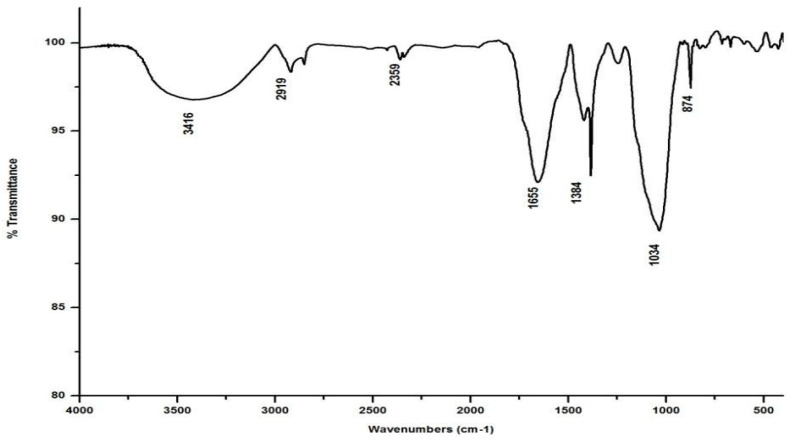
FTIR spectrum of the vermicompost generated from salvinia by *L. rubillus*.

**Figure 6:**
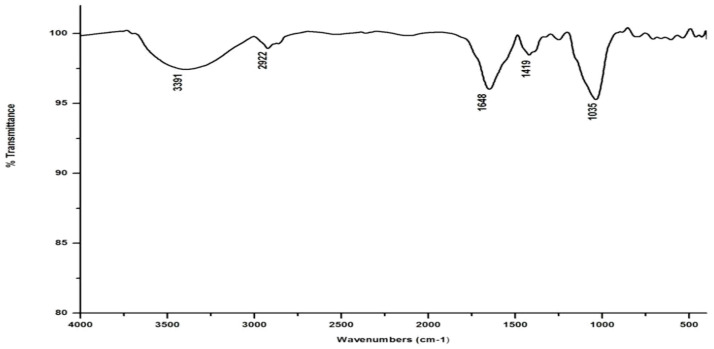
FTIR spectrum of the vermicompost generated from salvinia by *D. willsi*.

In marked contrast to the spectrum of the parent substrate, the spectra of all its vermicomposts reveal remarkable changes in the locations and intensities of peaks and troughs (Figure 3, Figure 4, Figure 5 and Figure 6). The peaks occurring in the 3100–3600 cm^−1^ wavelength range show that there is a significant reduction in the phenolic and alcoholic content of salvinia as it undergoes vermicomposting, which thereby reduces the concentrations of chemicals that likely induce its allelopathy. In contrast to the FTIR spectrum of salvinia, which has 92.5 percent of transmittance at the 3419 cm^−1^ peak, a slight increase in the transmittance (96–97%) was detected in all the vermicasts at 3391–3417 cm^−1^. The extent of the increase in transmittance is greatest in the case of *D. willsi*, followed by *L. rubillus, P. sansibaricus*, and *E. andrei*. However, considering the narrow range of transmittance by the vermicompost from the four earthworm species, it is clear that all four are capable of degrading phenols in a similar way, thereby exerting a similarly destructive effect on the weed’s allelopathy. It is also worth noting that in the vermicomposts’ FTIR, the bands occurring in the 2900–2800 cm^−1^ wavelength range are much shallower than the corresponding peaks in the salvinia FTIR. The peak at 1738 cm^−1^ is altogether absent in all of the vermicomposts’ spectra. This depicts the rigorous breakdown of lignin and the associated aliphatic compounds present in salvinia during its vermicomposting [35]. Previous research by Lim and Wu [36] led to similar observations; they found a reduction in band heights in the 3100–3600 cm^−1^, 2921 cm^−1^, and 2852 cm^−1^ regions in vermicomposts derived from palm-oil-mill residues using the earthworm species *Eisenia fetida*, when compared to the control.

Compared to the peak at 1634 cm^−1^ in the salvinia FTIR, a shift towards higher frequencies and greater intensity is manifested by all of the vermicomposts. Whereas the salvinia FTIR shows 95% transmittance at 1634 cm^−1^, all of the vermicomposts show 91–95.5% transmittance in the 1648–1655 cm^−1^ range. The increase in extent of absorption of the incident reduction follows the order *P. sansibaricus > E. andrei > L. rubillus > D. willsi*. This may be due to a shift in the structure of the polymers from crystalline to amorphous and the concomitant breakdown of lignin in the course of vermicomposting [37]. Barring the FTIR of the *D. willsi -* derived vermicompost, a sharp peak emerges around 1385 cm^−1^ in the FTIR of the vermicomposts of the other three species, which may be due to N-O stretching. This could indicate an enhancement in the nitrogenous compounds present in the vermicompost.

Vermicomposting appears to cause biodegradation of not only carbohydrates and hemicelluloses, but also a fraction of the lignin present in salvinia. These substances seem to be converted into polysaccharides. As a consequence, the bands in the 1000–1100 cm^−1^ range shift toward higher frequencies. Whereas salvinia has 95% transmittance at 1021 cm^−1^, all the vermicasts have transmittance in the 87.5–5% range at 1033–1035 cm^−1^. The intensity of the peak increases in the following order: *P. sansibaricus > L. rubillus > E. andrei > D. willsi.*

From the afore mentioned results, it can be seen that all four species of earthworm, epigeic as well as anecic, appear to have effected a breakdown of those chemicals in salvinia which are responsible for its intransigence and have introduced plant- and soil-friendly features to the resulting vermicomposts. Notably, this influence appears to be similar in nature and extent for all four earthworm species. Even when there were variations, they were only minor.

### 3.3. Thermogravimetric Analysis

**Figure 7:**
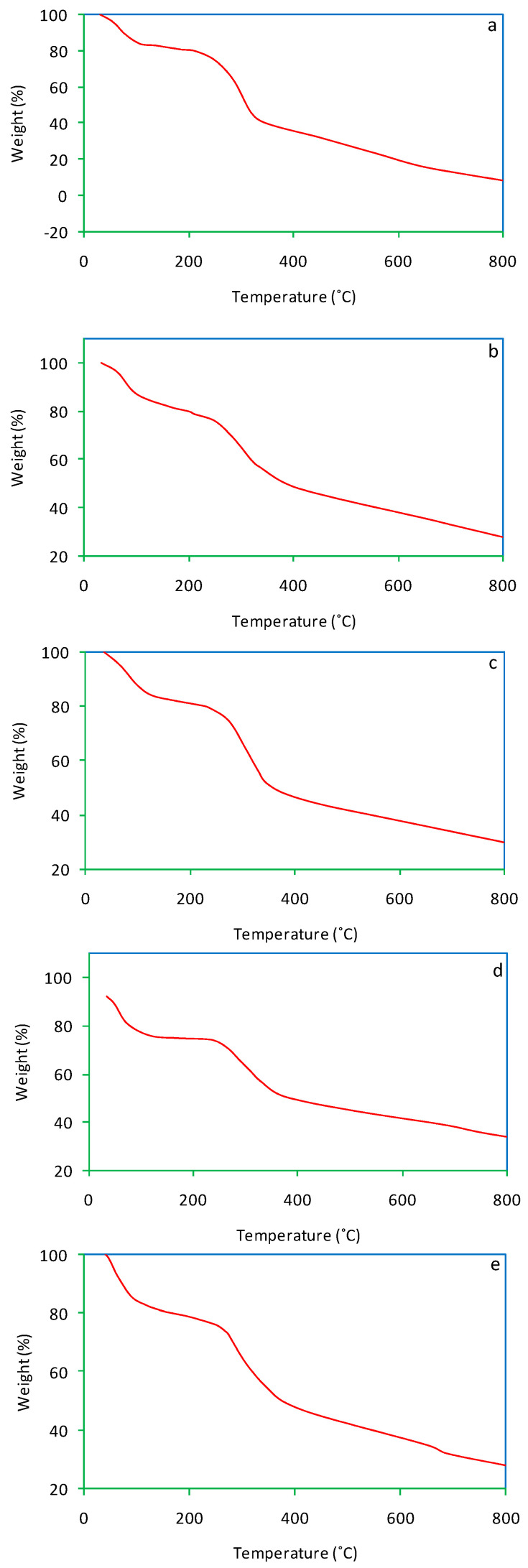
TG curves of salvinia (**a**) and of the vermicomposts produced by *E. andrei* (**b**), *P. sansibaricus* (**c**), *L. rubillus* (**d**), and *D. willsi* (**e**).

Thermogravimetric (TG) curves of salvinia and its vermicomposts are presented in Figure 7. For salvinia, a mass loss of 93.1% was recorded. In comparison, much less mass loss was suffered by the vermicomposts: 72%, 70%, 71.5%, and 72.3%, pertaining to *E. andrei, P. sansibaricus, L. rubillus,* and *D. willsi*, respectively. During previous research, Ganguly and Chakraborty [35] observed a mass loss of 80% and 71% in primary and secondary paper mill sludge vermicomposted by the earthworm *Perionyx excavatus*.

As the TG curves show, dehydration occurred in the temperature range 60–150 °C, followed by substrate disintegration when the temperature was raised beyond 200 °C. This happened to both salvinia and its vermicomposts, but in the vermicomposts the extent of mass loss was lesser than in the parent substrate [38,39]. This is reflective of the mineralization of organic matter caused by vermicomposting.

### 3.4. The Patterns of the Differential Scanning Calorimetric (DSC) Analysis

**Figure 8:**
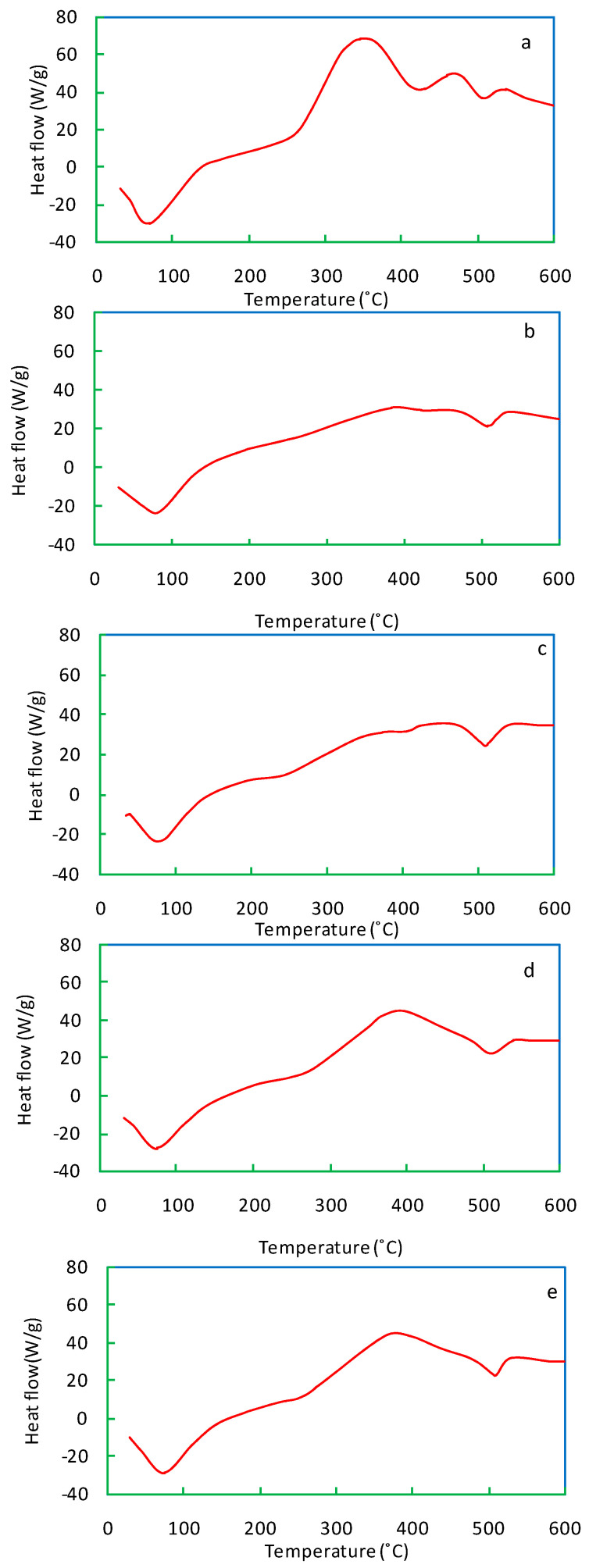
DSC curves of salvinia (**a**) and of the vermicomposts produced by *E. andrei* (**b**), *P. sansibaricus* (**c**), *L. rubillus* (**d**), and *D. willsi* (**e**).

The DSC curve of salvinia reveals two peaks, both exothermic (Figure 8). Carbohydrates and cellulose contained in salvinia may have led to the first peak in the 300–350 °C range. The second peak, occurring in the 450–500 °C range, may be due to lignin and phenols. In comparison, both of the exothermic peaks in the vermicomposts are visibly shallower (Figure 8b–e). The exothermic peaks lessen in accordance with the following order: *E. andrei > P. sansibaricus > L. rubillus > D. willsi.* These findings indicate that in the course of vermicomposting salvinia, there is a breakdown of its constituent simple carbohydrates, aliphatic compounds, and aromatic compounds (such as lignin and phenols) [40,41].

### 3.5. Findings of the Scanning Electron Microscopy

**Figure 9:**
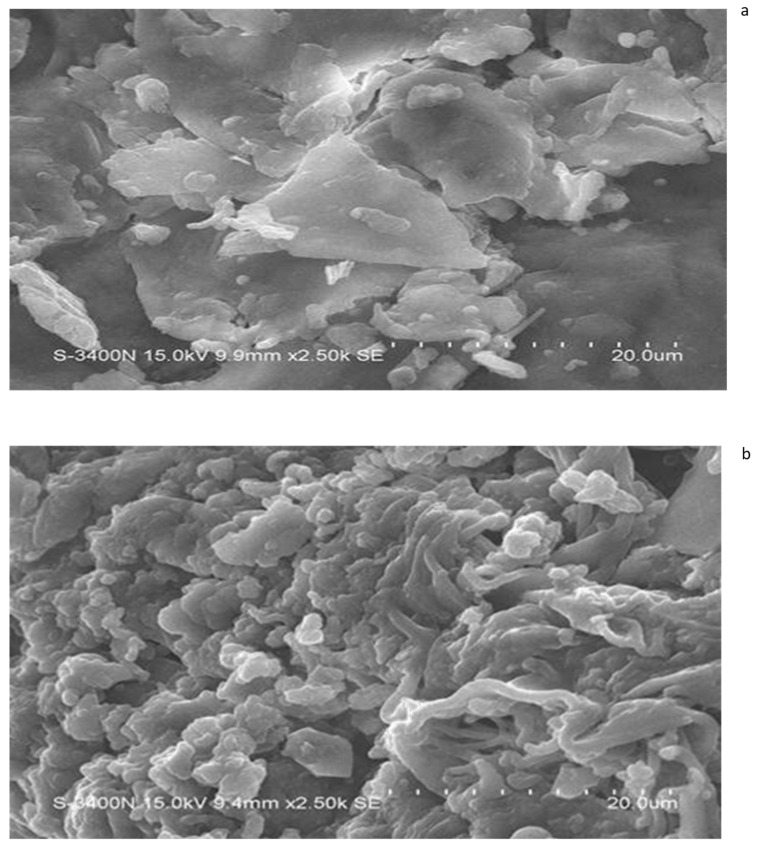
SEM images of salvinia (**a**) and of the vermicompost of *P. sansibaricus* (**b**).

The scanning electron micrographs (SEMs) of salvinia biomass indicate its relatively contiguous and robust structures—as expected from a very hardy and resilient weed (Figure 9). On the other hand, the SEM micrographs of the vermicomposts made by each of the four species of earthworm present a disaggregated and withered ambiance. This is exemplified by the micrograph of the *P. sansibaricus* vermicompost (Figure 9b). Similar findings were previously reported by Unuofin and Mnkeni [42], Lim and Wu [36], and Ganguly et al. [43]. Lim and Wu [36] reported a more fragmented nature of vermicompost compared to the long fibers present in palm oil mill by-product. Likewise, Ravindran et al. [44] observed significant alteration in the surface morphology of vermicompost derived from solid waste produced by the leather industry, compared to the initial substrates.

Lim et al. [45] indicated that when earthworms feed upon biomass, they grind it with their gizzard before ingesting it. The animals also release their gut microorganisms, enzymes, and hormones on the surfaces of the ingested biomass fragments to facilitate their biodegradation in the course of their passage through the earthworm gut [29,46,47,48,49,50]. Evidently, the disaggregation caused by this process is what is seen highlighted in the micrographs.

## 4. Summary

This paper reports several studies conducted to see the nature and extent of the transformations that occur when the highly ligninous and allelopathic weed, salvinia (*Salvinia molesta*), is converted to vermicompost by different species of earthworms. The aim of these studies was also to see whether the nature and extent of these transformations are common across different earthworm species, or vary from species to species. The studies were supported by UV-visible spectrophotometry, Fourier-transform infrared (FTIR) spectrometry, thermogravimetry, differential scanning calorimetry, and scanning electron microscopy.

It was observed that the C:N ratio of salvinia vermicomposts generated from each of the four epigeic and anecic earthworms was almost three times less than the C:N ratio of salvinia itself. Furthermore, and independent of the concerned earthworm species, vermicomposting caused a reduction in the phenol and lignin content of salvinia. TGA and DSC pertaining to vermicomposts of all the earthworm species indicated net mineralization as well as breakdown of simpler compounds (such as carbohydrates) and complex aromatic compounds (such as lignin). SEM micrographs confirmed that all four species caused the parent substrate to become extensively fragmented and withered in the course of vermicomposting.

The results show that, independent of which earthworm species is deployed, direct vermicomposting of salvinia carried out as per the process developed by the authors leads to a benign, highly stabilized, and potent organic fertilizer—without the need for any supplementation with animal manure or other substances. These findings present a process by which the millions of tons of presently unusable salvinia biomass generated every year can be converted into organic fertilizer. It also provides a means by which the harm to water bodies presently caused by salvinia infestation (as detailed in this paper) can be drastically reduced.

## Data Availability

All the pertinent data has been included in the paper.
